# Reflections on nursing students’ fear and anxiety arising from clinical practicums*

**DOI:** 10.17533/udea.iee.v40n3e13

**Published:** 2023-02-14

**Authors:** Agostinho Antônio Cruz Araújo, Simone de Godoy, Carla Aparecida Arena Ventura, Ítalo Rodolfo Silva, Emerson Willian Santos de Almeida, Isabel Amélia Costa Mendes

**Affiliations:** 1 Master Student. University of São Paulo, Ribeirão Preto College of Nursing, Ribeirão Preto, São Paulo, Brazil Email: agostinhocruz@usp.br Universidade de São Paulo Brazil agostinhocruz@usp.br; 2 Asociate Professor. University of São Paulo, Ribeirão Preto College of Nursing, Ribeirão Preto, São Paulo, Brazil Email: sig@eerp.usp.br Universidade de São Paulo Brazil sig@eerp.usp.br; 3 Full Professor. University of São Paulo, Ribeirão Preto College of Nursing, Ribeirão Preto, São Paulo, Brazil Email: caaventu@gmail.com Universidade de São Paulo Brazil caaventu@gmail.com; 4 Asociate Professor. Federal University of Rio de Janeiro, Macaé, Rio de Janeiro, Brazil Email: italoufrj@gmail.com Universidade Federal do Rio de Janeiro Brazil italoufrj@gmail.com; 5 Doctoral Student. University of São Paulo, Ribeirão Preto College of Nursing, Ribeirão Preto, São Paulo, Brazil Email: emersonwillian@usp.br Universidade de São Paulo Brazil emersonwillian@usp.br; 6 Emeritus Profesor, University of São Paulo, Ribeirão Preto College of Nursing, Ribeirão Preto, São Paulo, Brazil Email: iamendes@usp.br. Corresponding author Universidade de São Paulo Brazil iamendes@usp.br

**Keywords:** Anxiety, Fear, Nursing, Nursing Education, Students, Nursing., Ansiedad, Miedo, Enfermería, Educación en Enfermería, Estudiantes de Enfermería., Ansiedade, Medo, Enfermagem, Educação em Enfermagem, Estudantes de Enfermagem

## Abstract

**Background.:**

Anxiety and fear are emotional responses that may emerge when individuals anticipate threats. Undergraduate nursing students may experience feelings of hopelessness and anguish in the clinical learning experience, directly impacting their academic performance. This study aims to reflect upon the fear and anxiety faced by nursing students during clinical training.

**Synopsis of Contents.:**

Two thematic axes were focused: Students’ perception regarding preceptorship attitudes and positions; Relational teaching-learning processes and their influence on the students’ professional identity. Preceptors are expected to encourage the establishment and maintenance of good relationships in the collaborative network in which students are included, especially with the multi-professional health team, to have more comprehensive academic support.

**Conclusion.:**

The role and importance of each individual in academic training, such as students and professors, is emphasized, seeking to promote positive experiences in the teaching-learning process to enable undergraduate students to more effectively develop moral sensitivity and take responsibility for patient-centered care.

## Resumen

**Marco contextual.:**

La ansiedad y el miedo son respuestas emocionales que pueden surgir cuando las personas anticipan amenazas. Los estudiantes de pregrado en enfermería pueden experimentar sentimientos de desesperanza y angustia en la experiencia de aprendizaje clínico, lo que impacta directamente en su desempeño académico. Este estudio tiene como objetivo reflexionar sobre el miedo y la ansiedad que enfrentan los estudiantes de enfermería durante la formación clínica.

**Síntesis del contenido.:**

Se enfocaron dos ejes temáticos: la percepción de los estudiantes sobre las actitudes y posiciones de la preceptoría; Los procesos de enseñanza-aprendizaje relacionales y su influencia en la identidad profesional de los estudiantes. Se espera que los preceptores favorezcan el establecimiento y mantenimiento de buenas relaciones en la red colaborativa en la que se incluyen los estudiantes, especialmente con el equipo multiprofesional de salud, para tener un apoyo académico más integral.

**Conclusión.:**

Se enfatiza el papel y la importancia de cada individuo en la formación académica, como estudiantes y profesores, buscando promover experiencias positivas en el proceso de enseñanza-aprendizaje que permitan a los estudiantes de pregrado desarrollar más efectivamente la sensibilidad moral y asumir la responsabilidad del cuidado centrado en el paciente.

## Resumo

**Enquadramento.:**

A ansiedade e o medo são respostas emocionais que podem surgir quando os indivíduos antecipam ameaças. Estudantes de graduação em enfermagem podem vivenciar sentimentos de desesperança e angústia na experiência de aprendizagem clínica, impactando diretamente seu desempenho acadêmico. Este estudo tem como objetivo refletir sobre o medo e a ansiedade enfrentados por estudantes de enfermagem durante a formação clínica.

**Síntese do conteúdo.:**

Dois eixos temáticos foram enfocados: Percepção dos alunos sobre atitudes e posições de preceptoria; Processos relacionais de ensino-aprendizagem e sua influência na identidade profissional dos alunos. Espera-se que os preceptores incentivem o estabelecimento e a manutenção de boas relações na rede colaborativa em que os alunos estão inseridos, principalmente com a equipe multiprofissional de saúde, para ter um suporte acadêmico mais abrangente.

**Conclusão.:**

Ressalta-se o papel e a importância de cada indivíduo na formação acadêmica, como alunos e professores, buscando-se promover experiências positivas no processo ensino-aprendizagem para que os graduandos desenvolvam de forma mais efetiva a sensibilidade moral e assumam a responsabilidade pelo cuidado centrado no paciente.

## Introduction

A startup in the University of Oxford, United Kingdom, reports that anxiety disorders affect 3.8% of the world population, approximately 284 million people,(1) and for this reason, anxiety disorders are considered the disease of the 21st century.(2) These disorders comprise psychological comorbidities, including manifestations of anxiety and fear.(3) Anxiety is the anticipation of a real or imaginary threat, while fear is an emotional response to a situation an individual considers an imminent or future threat.(3) Regardless of an individual’s response, quality of life will be directly affected; there is evidence that anxiety negatively impacts academic performance.(4,5) In the context of nursing education, anxiety and fear compromise a student’s performance during clinical practicums.(6-8) 

Anxiety is identified based on a student’s perception regarding a programmed event, such as the beginning of clinical activities, while fear is perceived during the performance of technical, dialogical, or political tasks, practical classes, simulations, or supervised training. One descriptive study addressing nursing students reports that students might experience various situations with the potential to trigger anxiety and fear, such as fear of making mistakes, failing, not having sufficient knowledge, having a negative experience with their preceptors, not knowing how to act, and feeling uncomfortable when dealing with patients and their families. Furthermore, research shows that emotional responses are not restricted to the beginning of clinical activities; such responses are also found among already graduated individuals.(7)

A systematic review reports that anxiety and negative feelings, such as fear, adversely influence the performance of nursing students.(6) Studies addressing anxiety and fear among students are increasingly recognized, mainly because this is a recurrent condition that has become progressively evident during the covid-19 pandemic. Many students may manifest more significant anxiety and fear in these challenging times compared to regular contexts.(8) Therefore, our purpose is to explore fear and anxiety, considering that the manifestation of these conditions compromises a student’s learning process and possibly his/her professional future. Hence, our objective is to reflect upon the fear and anxiety faced by nursing students during clinical training. This reflection is based on two central themes: Students’ perception regarding preceptorship attitudes and positions; Relational teaching-learning processes and their influence on the students’ professional identity.

### Students’ perception regarding preceptorship attitudes and positions

Professors monitor undergraduate students in the practical field, except during supervised internships. Nurses employed by the institution selected for supervised internships are the preceptors of students. Thus, a need emerges in this context to compare the time spent and nature of the bond established between students and professors and students and preceptors. Studies show that anxiety and fear may emerge depending on preceptors’ attitudes.(9,10) For example, a cross-sectional study addressing nursing students at the University of Ontario, Canada, reports that one of the primary causes of anxiety among undergraduate students is the interaction with preceptors. Additionally, the students addressed by the above study reported that preceptors do not support them in the clinical environment.(10)

Fear and anxiety may cause either a positive or negative reaction on students. From a positive behavioural perspective, superior academic growth may result from stressors in a given discipline. On the other hand, students who negatively react to stressors become shy in the face of other group members, which compromises their learning and academic performance. Therefore, nursing students require monitoring during clinical training, considering that despite students’ autonomy, they cannot answer for the activities performed and require a preceptor. In addition, a qualitative study shows that students attending supervised training have the opportunity to develop managerial competencies and apply theoretical knowledge acquired during the undergraduate program into practice.(11) For this reason, a preceptor is fundamental in the teaching-learning process to encourage students to develop autonomy.

Preceptors are a reference for students in a hospital setting, providing guidance and performing procedures in their clinical practice.(12) However, it is necessary to reflect upon a preceptor’s role because the supervision of unqualified professionals may negatively impact a student’s training. The reason is that students may learn and incorporate habits that are incompatible with recommendations provided by professors and literature. From this perspective, there is a strategy of preceptorship in nursing to support students and preceptors. This strategy is composed of different roles/responsibilities: nursing student’s rights and duties during the internship; personal preceptor, the nurse who directly monitors the student; head preceptor, the nurse who supports students and preceptors to meet learning objectives; link teacher, the professor who participates in the internship planning and assesses students and preceptors; clinical teacher, the professor responsible for developing clinical competencies and supporting preceptors and the link teacher. However, implementing this strategy is challenging, especially regarding the length and improvement of students in each context.(12) 

Trust, reassurance, moral courage, and moral sensitivity are essential in any interpersonal relationship but require special attention and should be encouraged in practice settings. In addition, preceptors should encourage students to develop and maintain a good relationship with the support network, especially with the multi-professional health team, to ensure greater support. Trusting the monitoring and guidance provided by preceptors and professors is essential. Thus, both must have qualified theoretical and practical knowledge to ensure students feel confident, which positively influences their performance. The five-component model,(12) nursing student, personal preceptor, head preceptor, link teacher, and clinical teacher would be the most appropriate in the tutorship and preceptorship process, considering that the larger a student’s support network, the more motivated and self-assured the student will be to face the difficulties that emerge during clinical practicums.

Feeling comfortable with preceptors also influences the students’ level of moral courage developed during the learning experience and how they assess ethics from the mentor’s perspective as a model to be followed or not. Moral courage is considered a virtue in the nursing field and plays a vital role in molding a nurse’s personal and professional development.(13) On the other hand, the responsibility during the teaching and learning process is not restricted to the educator, but also needs to consider the personal availability of the student. Each student has different personal and academic motivations. Thus, their productivity in the clinical environment varies according to the interest area, and is also influenced by the relationship with the other students and the professor. Therefore, considering a positive interpersonal relationship of work/learning with the tutor, the student can deal with difficulties in the clinical environment. Thus, instead of negative psychological results, the lived experiences can base learning, contributing to personal and academic growth. 

Considering the individual characteristics of each student, the role of emotions in morality was explored considering the moral sensitivity concept, defined as a perception that another person’s action may affect one’s well-being.(14) The conclusion is that moral sensitivity can help nurses deal with the various demands and opinions that emerge in the daily routine within a healthcare setting, a context in which emotions are usually underestimated when considering moral issues. The authors of the study above argue that we need to dose cognition and emotion; otherwise, we may commit the mistake of despising a crucial part of morality in exercising our care roles. They state that nurses are likely to adopt various ideal types of moral sensitivity when confronted with an ethical issue, and many ideal types of moral sensitivity are found in the health field, especially among nurses. However, the authors refer to individuals more driven by rationale than emotion or those overly zealous of institutional values or group rules, considering that when we ground ourselves on reasoning only, we “leave out an essential part of morality.” The authors above consider that having morality means behaving according to one’s understanding of the good and bad in harmony with one’s upbringing, education, religion, and other socialization processes.(15)

The concept of moral sensitivity makes us reflect upon the teaching-learning process and the style of professors and preceptors that can either promote or prevent a student from coping with fear and anxiety.(15) Professors and preceptors should encourage, contribute, respect the students’ individuality, and welcome students for choosing the profession instead of rejecting their choice, which hinders their development. Students need to be driven, nurtured, and encouraged to bloom and give their best to become leaders so that they will value their profession and themselves as excellent professionals at their community’s service. Therefore, those responsible for students, either professors or preceptors, should identify the students’ traits that may interfere in their development in a clinical setting and the construction of their professional identities, such as a self-demanding personality or making social comparisons, as each individual has a different level of confidence in performing tasks. Therefore, one should aim to decrease learning inequalities from the perspective of educational strategies. 

### Relational teaching-learning processes and their influence on the students’ professional identity 

Moral distress causes feelings of hopelessness.(16) Thus, we need to consider it in the training process of nursing students because, regardless of emotional manifestations, it may interfere in an individual’s growth and permanence in the profession.(17) Furthermore, moral distress is not restricted to an event and the time it occurs; instead, it is recurrent and may lead students to experience anguish.(18) Moral suffering can directly impact students' motivation to continue the program. As students experience situations of fear and anxiety in a clinical environment, and do not deal with these challenges, this can result in damage to their academic performance. Therefore, professors and preceptors need to pay attention to students when performing their activities, especially in a clinical setting. Whenever students face a situation that leads to mistakes or uncertainty during their practice, they may have second thoughts about pursuing a career in the nursing field and quit, even if having the potential to grow in the profession successfully. 

Nonetheless, a student should not be monitored and assessed based only on his/her individuality but also from a collective perspective.(17) The reason is that a student may perform better when alone than when in a group composed of peers, preceptors, or professors. Students are expected to develop and improve leadership during the academic program, and leadership is an essential characteristic in the nursing field because nurse leaders transform reality. Additionally, teamwork should be encouraged since the beginning of the program as nursing professionals are expected to lead the nursing staff and play a collaborative role in inter-professional teams. Clinical settings enable students to develop and improve their practical skills allied with theoretical knowledge. Training in this environment effectively contributes to one’s professional identity.(18) However, academic success is not only effectively fulfilling the disciplines’ requirements. On the contrary, intrinsic factors (e.g., being able to manage tasks, assume responsibilities and deal with setbacks, being motivated, and developing professionalism and communication skills) and extrinsic factors (e.g., the quality of teaching and the support provided by educators in this environment) influence a student’s performance.(19) From this perspective, a self-demanding personality and making social comparisons influence a student’s self-confidence during the training process. Stress experienced in the teaching environment may affect academic satisfaction and harm a student’s performance, causing disinterest and lack of motivation toward the training process.(20) 

Faced with dissatisfaction in the teaching environment, students may start demanding themselves to improve their performances or even seek perfection in clinical tasks. Unfortunately, this behaviour may compromise academic achievement and quality of life as students may spend time with academic tasks that should otherwise be dedicated to leisure and resting, in an attempt to achieve satisfaction and perform as well as their peers. Students must adequately administer their time dedicated to academic assignments and studies to deal with stressors during academic training (e.g., second doubting oneself or negativism). In addition, the university plays an essential role in promoting a reassuring environment when providing services to preserve and promote students’ physical and mental health. Thus, contributions to avoid more severe conditions, such as depression, are not restricted to students’ academic development but also their personal growth.(21)

In 1954, Leon Festinger introduced the expression “social comparison,” defined as a way to change one’s behaviour based on someone else’s skills.(22) Social comparison is highly likely to occur during one’s academic training. Students perform evaluative activities throughout the program and try to match other students’ performance, comparing each other’s achievements.(23) From this perspective, up to a certain degree, social comparison can positively influence a student’s performance, as they may identify aspects that can be improved. However, frequently assimilating other students’ behaviours will negatively influence one’s self-confidence.

Being self-demanding and social comparison is inevitable during the clinical training of nursing students. Hence, interventions are needed to help students deal with a lack of self-confidence, anxiety, and fear during their academic training. The earlier these characteristics are identified, and strategies are implemented to cope with them, the more likely students will improve their performance in academic activities, practical classes, and supervised training. 

In order to contribute to the international organizations’ goals in favor of health policies, especially with respect to the composition of the teams with the participation of nurses, it is important to stimulate the schools, as well as faculty, preceptors or technicians, to understand the need to embrace students who are interested in the profession. Therefore, policy makers must take into consideration the recommendations of the State of the World´s Nursing Report driving strategic investments to Nursing education, inspiring and retaining talents to perform well their role in society.(24) Globally, the different actors in Nursing education should use all investments to prepare the new generation of nurses well.(25) The private or public organizations which employ nurses, must value and retain these professionals, through multiple incentives that will reflect in the quality of the healthcare delivered.(24)

Thus, the relational processes are the foundation to interactive situations involving the learning-teaching process, but also interefering in the professional identity of nurses, as well as are the basis for a teaching offered in accordance to local, regional and global needs. In the case of nurses training, it is relevant to consider the data presented in the State of the World´s Nursing Report,(24) especially related to the deficit of 5.9 million nursing professionals in 2018. These issues discussed in the end of the first decade of the 21th century, and more often in the beginning of the actual decade, are based on evidences that increased the concern of international organizations and governments and enabled the development of the Nursing Now Campaign. In this perspective, this reflection is centered on the fear and anxiety faced by Nursing students, a theme that must be considered by the institutions that are responsible for these professionals training. This scenario of a high deficit asks for behaviors directed to inspire, stimulate, retain and value these human resources. 

## Conclusion

These reflection discussions were based on the specificities of the roles played by professors/preceptors, students, and the organizations involved. In summary, we reiterate the importance of these actors to construct positive teaching-learning experiences and encourage nursing students to develop their moral sensitivity and take responsibility for patient-centered care individually and jointly more effectively.
